# Flavonoids as Inhibitors of VEGFR2 Signaling: Structural Insights for the Development of Safer Anti-Angiogenic Therapies

**DOI:** 10.3390/ijms27083605

**Published:** 2026-04-18

**Authors:** Andrew Yim, Jianming Lu, Wei Wen

**Affiliations:** Department of Surgery, City of Hope National Medical Center, Duarte, CA 91010, USA; anyim@coh.org (A.Y.); jlu@coh.org (J.L.)

**Keywords:** angiogenesis, VEGF/VEGFR, flavonoid, structure–activity relationship

## Abstract

Vascular endothelial growth factor (VEGF) is a key regulator of angiogenesis and an established therapeutic target in diseases such as cancer and ocular disorders. However, long-term use of most current anti-VEGF agents is often limited by their associated side effects, including hypertension, bleeding, and gastrointestinal complications. These limitations have stimulated interest in naturally occurring VEGF inhibitors derived from dietary sources, which may offer safer alternatives due to their favorable safety profiles. In this study, we investigated shared structural features of potent VEGFR2 inhibitors, focusing on naturally derived polyphenols. Polyphenols representing multiple structural subclasses were evaluated for their ability to inhibit VEGFR2 kinase activity using an in vitro kinase assay, to suppress VEGF-induced phosphorylation of VEGFR2 and downstream MAPK signaling in endothelial cells by Western blot, and to reduce VEGF-stimulated endothelial cell proliferation. Across all assays, flavonoids with strong VEGFR2 inhibitory activity displayed consistent structural characteristics, including the number and specific positioning of hydroxyl groups on the A- and B-rings, as well as specific structural elements of the C-ring. Our findings provide a strong foundation for further structure–activity relationship (SAR) studies and facilitate identification of key molecular determinants required for VEGFR2 inhibition. Elucidation of these structural patterns may contribute to the development of more effective and safer angiogenesis inhibitors with reduced adverse effects.

## 1. Introduction

Angiogenesis, the process of new blood vessel formation from pre-existing vasculature, plays a pivotal role in tumor progression. This process is tightly regulated through a delicate balance of pro-angiogenic and anti-angiogenic factors. Tumor angiogenesis involves multiple steps, each presenting potential targets for therapeutic intervention. Angiogenesis inhibitors, which suppress blood vessel formation, have emerged as a promising class of drugs in cancer treatment [1,2,3].

Among the many pro-angiogenic factors, vascular endothelial growth factor (VEGF) is one of the most critical and specific regulators of angiogenesis. It induces angiogenesis via binding to its two receptor tyrosine kinases expressed on endothelial cells, VEGF receptor 1 (flt1/VEGFR1) and VEGF receptor 2 (KDR/flk1/VEGFR2). Binding of VEGF to these receptors induces conformational changes, leading to receptor dimerization and autophosphorylation of their tyrosine residues, which subsequently triggers the formation of new blood vessels. In addition to VEGFR-mediated signaling, other receptor families also contribute significantly to the regulation of angiogenesis, including fibroblast growth factor receptors (FGFRs), platelet-derived growth factor receptors (PDGFRs), and angiopoietin receptors (Tie1/Tie2) [1,2,3]. Inhibiting VEGF activity often results in the suppression of tumor growth [4,5]. Various strategies to inhibit VEGF activity have been evaluated in preclinical and clinical studies. These strategies include antibodies targeting VEGF ligands or receptors, soluble receptors that sequester ligands, and small molecule inhibitors that block kinase activity [6]. Nearly all anti-angiogenic drugs approved by the US Food and Drug Administration (FDA) target the VEGF pathway [6,7].

However, many of these agents are associated with side effects, such as hypertension, bleeding, and gastrointestinal perforation, which limit their long-term use [8]. A diet-based approach to reducing VEGF activity offers an attractive alternative, given its established safety in human use. Polyphenols, particularly flavonoids, are well-known beneficial active components found in natural food products [9,10,11,12,13,14,15,16]. These compounds are abundant in a wide variety of foods and beverages, including tea, chocolate, fruits, vegetables, beans (such as soy), spices, and red wine. The average dietary intake of polyphenols in the general population is estimated to be around 900 mg per day. Meta-analyses of both epidemiological and intervention studies have demonstrated an inverse relationship between the consumption of flavonoids and other polyphenols and the risk of various chronic diseases, including cancers, type II diabetes, cardiovascular disease, and more [15,17,18,19,20,21,22,23,24].

Recent research has highlighted that polyphenols, particularly flavonoids, extracted from various plant sources such as soy, berries, pomegranate, grape seeds, and green tea, exhibit potent anti-angiogenic properties. These compounds exert their effects by interfering with key angiogenesis signaling pathways, such as the VEGF/VEGFR pathway [11,25,26,27,28,29,30,31,32].

The current study aims to evaluate the inhibitory effects of a wide range of flavonoids on VEGFR2 kinase activity, with the goal of identifying structural similarities among the most potent inhibitors.

## 2. Results

### 2.1. Effects of Flavonoids on VEGFR2 Kinase Activity

Polyphenols are broadly classified into four main groups, flavonoids, phenolic acids, lignans, and stilbenes, with flavonoids being one of the most extensively researched groups. Flavonoids are characterized by a C6−C3−C6 skeleton, which consists of two benzene rings (designated as rings A and B) connected by a three-carbon bridge that often forms a heterocyclic pyran ring, known as the C-ring. Based on the chemical structure of this central C-ring, flavonoids are further subdivided into subclasses such as flavonols, flavones, anthocyanins, and catechins [33,34]. To better understand the relationship between structure and activity, an in vitro VEGFR-2 tyrosine kinase assay was conducted to identify which flavonoids most effectively inhibit VEGFR2 kinase activity following incubation with various flavonols, flavones, anthocyanidins, catechins, and other related compounds.

**Figure 1:**
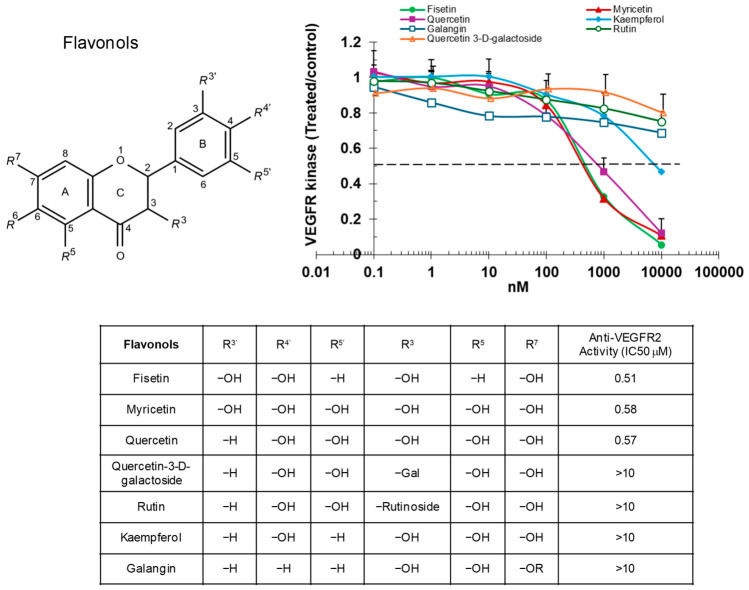
Flavonols inhibit VEGFR2 kinase activity. VEGFR2 was incubated with increasing concentrations of flavonols, and substrate phosphorylation was measured using an enzyme-linked immunosorbent assay (ELISA). Data are expressed as a ratio relative to the vehicle-treated control.

Among the flavonols tested, fisetin, myricetin, and quercetin exhibited similar inhibitory effects on VEGFR2 kinase activity, with IC_50_ values of approximately 0.5 μM (Figure 1). Interestingly, replacing the hydroxyl group at the R3 position of quercetin in the C-ring with a galactose (as in quercetin-3-D-galactoside) or rutinoside (as in rutin) group led to a significant increase in IC_50_, highlighting the critical role of the hydroxyl group at this position. In addition, kaempferol’s inhibitory activity was markedly diminished, with a more than 20-fold increase in IC_50_ when both hydroxyl groups at positions R3′ and R5′ in B-ring were absent. Galangin, which lacks hydroxyl groups at R3′, R4′, and R5′, exhibited very weak inhibitory activity. Fisetin, quercetin and myricetin, all possessing at least two hydroxyl groups on the B-ring, had comparable inhibitory activities. Taken together, these findings indicate that the kinase inhibition activity of flavonols is primarily driven by the hydroxyl group at the R3 position, as well as by the presence of at least two hydroxyl groups at the R3′, R4′ and R5′ positions of the B-ring.

**Figure 2:**
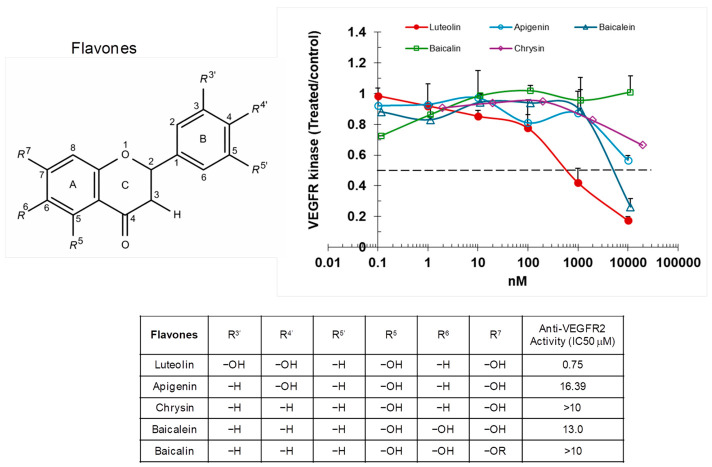
Flavones inhibit VEGFR2 kinase activity. VEGFR2 was incubated with varying concentrations of flavones, and substrate phosphorylation was measured by ELISA. Results are presented as ratios relative to the vehicle-treated control.

Out of flavones evaluated, luteolin, which has two hydroxyl groups on the B-ring, exhibited the lowest IC_50_ value (~0.75 μM; Figure 2). This potency exceeded that of less hydroxylated flavones like apigenin, containing only one B-ring hydroxyl, as well as flavones without B-ring hydroxyls such as chrysin, baicalein, and baicalin. Chrysin, lacking the R6 hydroxyl on the A-ring, and baicalin, in which the R7 hydroxyl is replaced by a glucuronide moiety, both exhibited IC_50_ values beyond the measurable range (>10 µM), whereas baicalein, containing both hydroxyls, showed a measurable IC_50_ of ~7 µM, highlighting the critical contribution of A-ring hydroxyl groups to inhibitory activity. Collectively, these findings highlight the critical role of the hydroxyl groups on A- and B-rings in modulating the inhibitory potency of flavones against VEGFR2 kinase activity.

**Figure 3:**
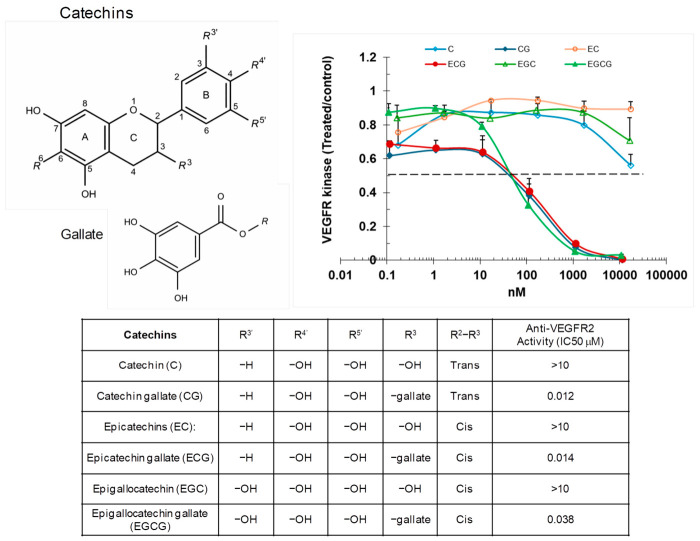
Catechins inhibit VEGFR2 kinase activity. VEGFR2 was incubated with varying concentrations of catechins, and substrate phosphorylation was evaluated using ELISA. Data are presented as ratios relative to the vehicle-treated control.

Among all the catechins tested, those containing a gallate group at the R3 position of the C-ring exhibited notably low IC_50_ values, indicating strong inhibitory activity (Figure 3). Specifically, catechin gallate (CG), epicatechin gallate (ECG), and epigallocatechin gallate (EGCG) demonstrated IC_50_ values of 0.012 μM, 0.014 μM, and 0.038 μM, respectively, highlighting their potent ability to inhibit VEGFR2 kinase. The presence of the gallate group at the R3 position of the C-ring appears to significantly enhance their inhibitory potency compared to other catechins. Gallic acid or methyl gallate, which lacks the catechin backbone and is simply a phenolic compound, exhibited a much higher IC_50_, demonstrating that the structural complexity and the catechin core structure play a crucial role in modulating the compound’s effectiveness. This suggests that the combination of the catechin framework with the gallate group at the R3 position of the C-ring is essential for maximizing the inhibitory activity of these compounds.

**Figure 4:**
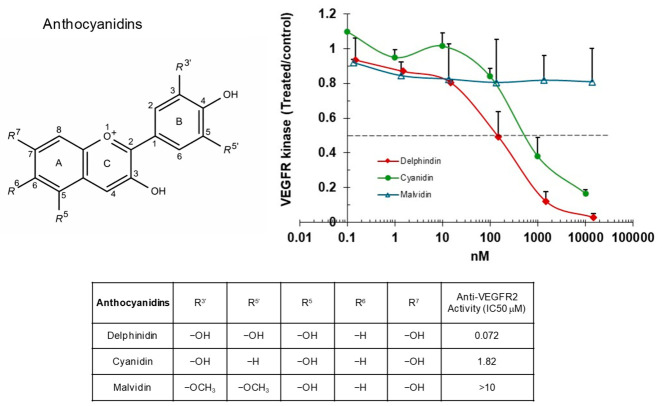
Anthocyanidins inhibit VEGFR2 kinase activity. VEGFR2 was incubated with varying concentrations of anthocyanidins, and substrate phosphorylation was measured by ELISA. Data are expressed as ratios relative to the vehicle-treated control.

The IC_50_ value for delphinidin was approximately 0.072 μM. Cyanidin, which lacks a hydroxyl group at the R5′ position of the B-ring, exhibited a higher IC_50_ of 1.82 µM. Furthermore, replacing the hydroxyl groups at both the R3′ and R5′ positions of the B-ring with methoxy (−OCH_3_) groups in malvidin resulted in IC_50_ values beyond the measurable range (>10 µM) (Figure 4). These results indicate that the presence of hydroxyl groups at specific positions of the B-ring is associated with stronger inhibitory activity of anthocyanidins.

**Figure 5:**
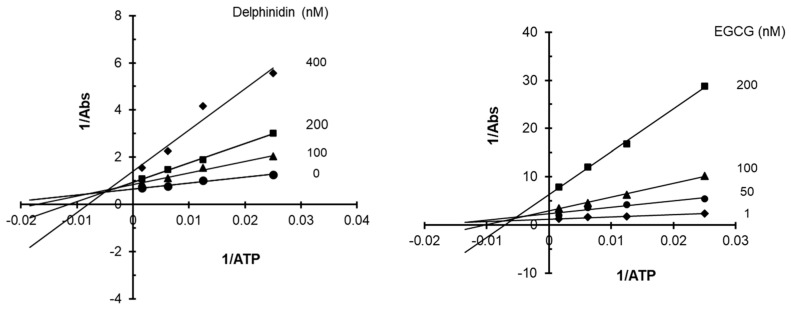
Lineweaver–Burk plot illustrating the inhibition of VEGFR2 by delphinidin or EGCG. VEGFR2 was incubated with increasing concentrations of adenosine triphosphate (ATP) in the presence of varying concentrations of delphinidin or EGCG.

To better understand the biochemical mechanism underlying the inhibition of VEGFR2, we assessed the effects of delphinidin, a prominent anthocyanidin, and EGCG, a major catechin, on VEGFR2 kinase activity at various ATP concentrations. By measuring the kinase’s activity across these varying concentrations, we generated a Lineweaver–Burk plot to assess the effects of the compounds on the enzyme kinetics. The resulting data showed clear alterations in both the apparent *x*-axis and *y*-axis intersections as the concentrations of delphinidin and EGCG were increased, indicating both delphinidin and EGCG may act as mixed inhibitors of VEGFR2 kinase activity. This type of inhibition indicates that both delphinidin and EGCG likely interact with VEGFR2 in a way that simultaneously affects both VEGFR2’s affinity for ATP and its kinase activity (Figure 5).

Taken together, these findings highlight the importance of hydroxylation at specific sites for effective VEGFR2 kinase inhibition, providing insight into how different structural substitutions can influence a compound’s potency.

### 2.2. Effects of Flavonoids on VEGFR2 Signaling on Endothelial Cells

To validate the results from the in vitro kinase activity assays, we examined the effects of selected polyphenols on the VEGFR2-mediated signaling pathway in endothelial cells using Western blot analysis. MAPK/ERK2, a key signaling pathway activated upon VEGFR2 stimulation, plays a crucial role in supporting endothelial cell proliferation, survival, and migration [4]. Representatives from the flavonol, flavone, anthocyanin, and catechin subclasses were incubated with HUVECs and then stimulated with VEGF for 5 min. The phosphorylation of VEGFR2 and p42/44 MAPK was subsequently assessed by Western blot.

**Figure 6:**
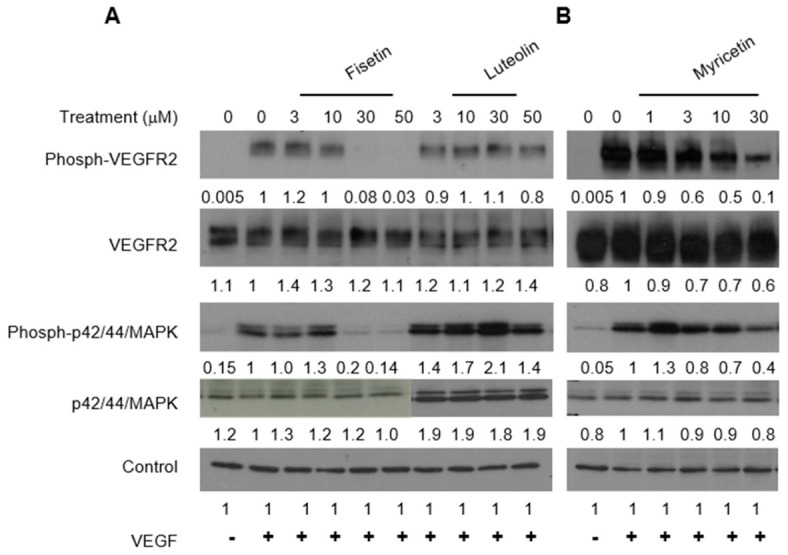

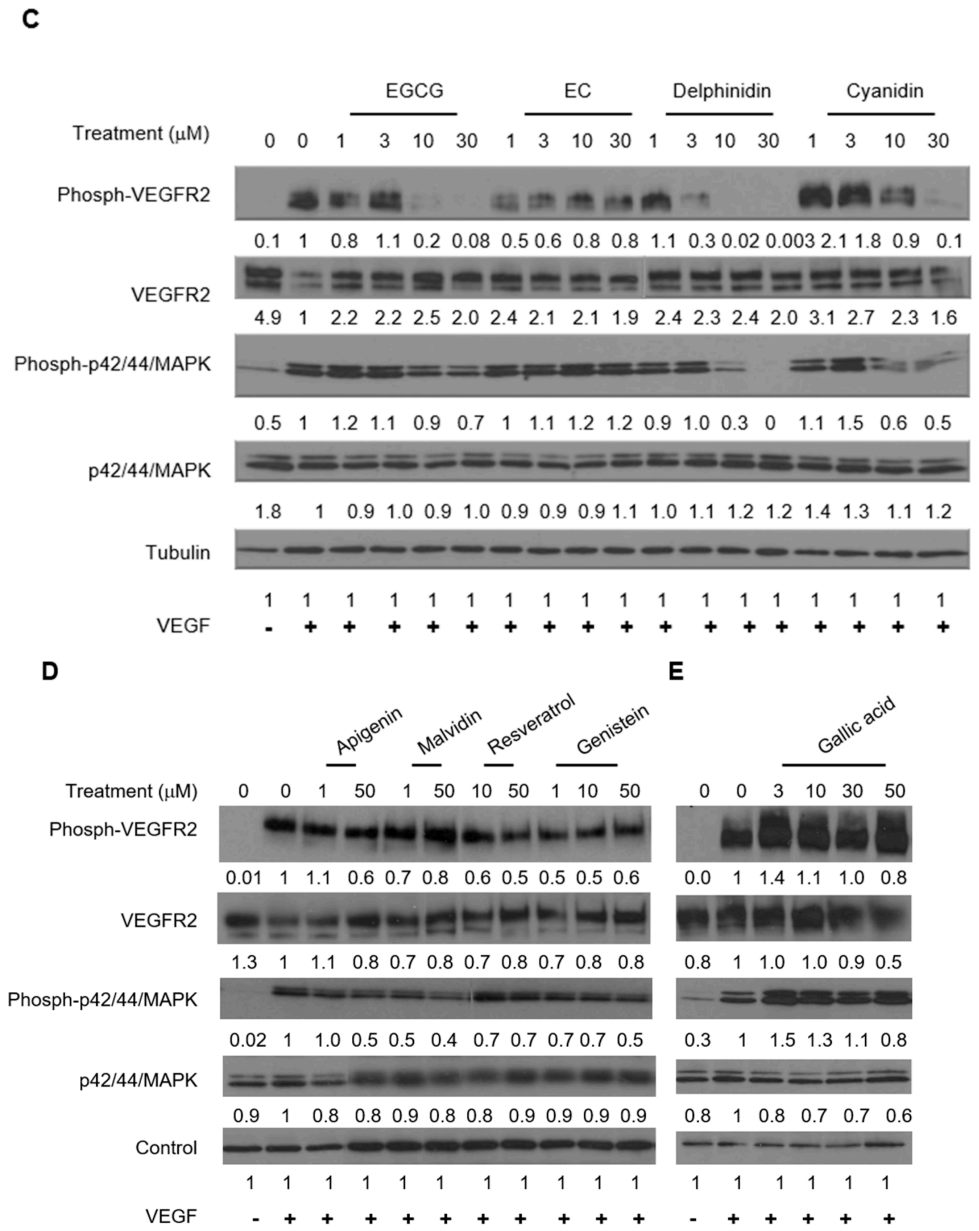
Flavonoids inhibit VEGFR2 signaling in endothelial cells. Quiescent HUVECs were treated with the indicated compounds and stimulated with VEGF (100 ng/mL, 5 min). Panels show (**A**) fisetin and luteolin; (**B**) myricetin; (**C**) EGCG, EC, delphinidin, and cyanidin; (**D**) apigenin, malvidin, resveratrol, and genistein; (**E**) gallic acid. Lane 1: no VEGF (0 VEGF); Lane 2: VEGF only (0 flavonoid); subsequent lanes: increasing flavonoid concentrations (based on prior kinase assays). Phosphorylated and total VEGFR2 and p44/42 MAPK were analyzed by Western blot. β-Actin (panel **B**) or β-tubulin (others) served as loading controls. Band intensities were quantified, normalized to loading controls, and are shown below each band. Signal variation in panels (**D**,**E**) reflects samples prepared on different days with minor protein differences.

Both VEGFR2 and p42/44 MAPK were phosphorylated upon the addition of VEGF to HUVECs (Figure 6). Pretreatment with fisetin and myricetin significantly inhibited VEGF-induced phosphorylation of both VEGFR2 and MAPK in a dose-dependent manner, without affecting their overall expression levels (Figure 6A,B), which is consistent with the in vitro kinase assay findings. In contrast, apigenin and malvidin, both of which exhibited low activity in the in vitro assays, had minimal effects on VEGFR2 and p42/44 MAPK phosphorylation (Figure 6D). Additionally, genistein, a well-known isoflavone, and resveratrol, a well-known stilbene, showed little to no activity in Western blot analysis, in line with their lack of significant activity in the in vitro assays). Although luteolin demonstrated IC_50_ values comparable to those of fisetin and myricetin in the in vitro kinase assay (Figure 1 and Figure 2), its inhibition of VEGFR2 and p42/44 MAPK phosphorylation was weaker, possibly due to limited membrane permeability, rapid intracellular metabolism (e.g., glucuronidation or sulfation), and active efflux via transporters such as P-glycoprotein. Similar discrepancies have been reported in the literature, underscoring the challenge of translating biochemical potency to cellular effects. Potential strategies to enhance cellular activity, such as structural modifications (methylation, prodrugs) or delivery systems (nanoparticles, liposomes), may improve efficacy and support the development of safer anti-angiogenic therapies [9,10,11,12,13,14,15].

EGCG significantly blocked VEGF-induced phosphorylation of VEGFR2 and p42/44 MAPK in a dose-dependent manner, while epicatechin (EC), which lacks the gallate group at the R3 position, had minimal effect on VEGFR2 and p42/44 MAPK phosphorylation (Figure 6C). Similarly, gallic acid alone showed little impact on VEGFR2 and p42/44 MAPK phosphorylation (Figure 6E). These findings are consistent with the results from the in vitro kinase assay.

Delphinidin effectively inhibited VEGF-induced phosphorylation of VEGFR2 and p42/44 MAPK at concentrations as low as 3 μM, whereas cyanidin required approximately 10 μM to achieve similar inhibition, further supporting the in vitro kinase assay data (Figure 6C).

**Figure 7:**
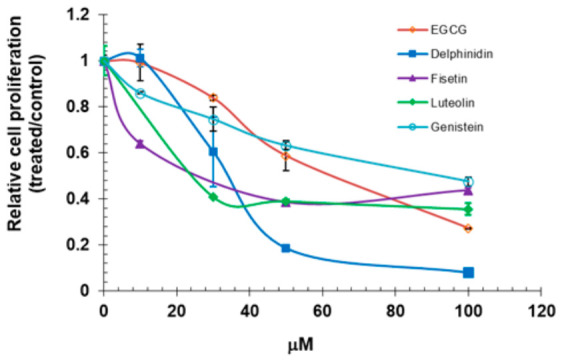
Flavonoids inhibit endothelial cell proliferation. HUVECs were treated with varying concentrations of flavonoids, stimulated with VEGF, and cell number was assessed after 48 h. Data are presented as ratios relative to the vehicle-treated control.

We next evaluated the effect of polyphenols on VEGF-induced endothelial cell proliferation (Figure 7). Endothelial cells were pretreated with various concentrations of selected polyphenols and then stimulated with VEGF (100 ng/mL). Cell proliferation was assessed 48 h later. As shown in Figure 7, VEGF-induced HUVEC proliferation was markedly suppressed in the presence of the selected polyphenols. Delphinidin showed a stronger inhibitory effect compared to genistein, consistent with the results from the in vitro kinase assay. Overall, flavonoids had a weaker effect on proliferation than on VEGFR2 and MAPK phosphorylation, reflecting differences in assay sensitivity and mechanisms. While EGCG and delphinidin reduced phosphorylation, this alone may be insufficient to fully inhibit proliferation, which is regulated by multiple pathways. Western blot captures early signaling (5 min VEGF), whereas proliferation reflects longer term responses. High flavonoid concentrations may also cause nonspecific effects, so results at higher doses should be interpreted with caution.

Taken together, representatives from the flavonol, flavone, anthocyanin, and catechin subclasses exhibited similar inhibition of VEGFR2 signaling and endothelial cell proliferation. The consistency of the results across the in vitro kinase assay, VEGFR2 signaling analysis by Western blot, and cell proliferation assays reinforces their significance.

## 3. Discussion

A diverse range of natural products have been identified as inhibitors of angiogenesis, often targeting the VEGF signaling pathway, and have been investigated for their potential as anti-cancer agents [9,10,11,13]. In this study, we quantitatively evaluated the effects of flavonoids from different structural subclasses on VEGFR kinase activity using an in vitro kinase assay. Additionally, we examined the phosphorylation of VEGFR2 and downstream MAPK/ERK2 signaling in endothelial cells via Western blot analysis. These results provide valuable insights into the structural characteristics of flavonoids that contribute to VEGFR inhibition.

The anti-angiogenic activity of flavonoids has been extensively investigated over the past two decades, with numerous studies demonstrating their ability to inhibit angiogenesis by targeting key signaling pathways, particularly those involving vascular endothelial growth factor (VEGF) and its receptors (VEGFRs) [25,33,35,36,37]. Flavonoids can interfere with angiogenesis through multiple mechanisms: by binding directly to VEGFRs and blocking activation [38]; by suppressing VEGF production through downregulation of its gene expression or its upstream regulators such as hypoxia-inducible factor 1-alpha (HIF-1α), a key transcription factor that controls VEGF expression under hypoxic conditions [39]. In addition, certain flavonoids can bind directly to VEGF itself, thereby blocking its interaction with VEGFRs and further inhibiting downstream signaling [40].

Flavonoids have been shown to act as kinase inhibitors by targeting the ATP-binding site [41,42]. The chromenone moiety, a key structural feature of flavonoids, mimics the adenine ring of ATP, enabling these compounds to effectively compete for binding within the kinase active site. In this study, our results demonstrated that flavonoids such as delphinidin and EGCG simultaneously reduce both VEGFR2’s affinity for ATP and its kinase activity (Figure 5). Moreover, the inhibitory effect of flavonoids on VEGFR kinase activity is strongly influenced by the number, position, and nature of hydroxyl (OH) groups.

Flavonols such as fisetin, myricetin, and quercetin, each with at least two B-ring hydroxyl groups, exhibited significantly greater VEGFR inhibition than less hydroxylated flavonols like kaempferol and galangin. Similar trends were observed in flavones and anthocyanidins. Among flavones, luteolin (two B-ring OH) inhibited VEGFR more strongly than apigenin (one OH) or flavones lacking B-ring hydroxyls (chrysin, baicalein, baicalin). Baicalin, a glycosylated baicalein derivative with R7 OH replaced by glucuronide, showed markedly reduced activity. Genistein, an isoflavone and structural isomer of apigenin, exhibited similar inhibition. In anthocyanidins, delphinidin (three B-ring OH) was more effective than cyanidin (one OH) or malvidin, where two hydroxyls are replaced by O-methyl groups. A-ring hydroxylation also influenced activity: baicalein (three A-ring OH) was more potent than apigenin or chrysin (two OH). These results highlight the critical role of specific hydroxylation patterns in VEGFR inhibition, The hydroxyl groups likely contribute to activity by forming hydrogen bonds with amino acid residues within the kinase’s active site.

Flavonoids are classified by the structure of their central C-ring, which is essential for their biological activity. The R3 hydroxyl group is particularly important; quercetin derivatives such as quercetin-3-D-galactoside and rutin, in which R3 is replaced by a sugar, show little inhibitory activity. Other C-ring features, including the C2=C3 double bond and R4 carbonyl, also contribute to inhibition. Although catechins and anthocyanidins lack these features, delphinidin has a lower IC_50_ than non-galloylated catechins, likely due to C-ring charge and additional double bonds. Galloylation at R3, as in catechin gallates and EGCG, significantly enhances inhibition. These results highlight the critical role of C-ring structure in VEGFR inhibition. In summary, the VEGFR inhibitory activity of flavonoids is strongly influenced by hydroxylation patterns on the A- and B-rings and structural features of the C-ring. These structure–activity relationships provide a framework for designing more potent flavonoid-based anti-angiogenic agents. Combined with their antioxidant, anti-inflammatory, and immunomodulatory effects, as well as favorable safety and cost profiles, flavonoids represent promising candidates for further development as anti-cancer therapeutics [15,24,43,44,45,46].

## 4. Materials and Methods

### 4.1. Chemicals

Flavonoids were purchased from Sigma-Aldrich (St. Louis, MO, USA), including fisetin, myricetin, quercetin, quercetin 3-D-galactoside, rutin, kaempferol, galangin, luteolin, apigenin, chrysin, baicalein, baicalin, cyanidin, delphinidin, malvidin, catechin hydrate, catechin gallate, epicatechin, epicatechin gallate, epigallocatechin, and epigallocatechin gallate. All flavonoids were prepared in DMSO at a stock concentration of 10 mM, then diluted to 10 μM, followed by a 1:10 serial dilution for the kinase assay.

### 4.2. In Vitro Kinase Assay

In vitro VEGFR2 tyrosine kinase activity was assayed using an ELISA kit (Sigma-Aldrich, St. Louis, MO, USA) as described previously [27,32]. Briefly, flavonoids at various concentrations were incubated with VEGFR2 (Upstate, Lake Placid, NY, USA, distributed by Sigma-Aldrich) in kinase reaction buffer containing Mn^2+^, Mg^2+^ and ATP in 96-well plates coated with a poly-Glu-Tyr substrate. Substrate phosphorylation was monitored using a phosphotyrosine-specific monoclonal antibody conjugated to HRP (Upstate, distributed by Sigma Aldrich). Results were expressed as ratio of control (DMSO treatment); IC_50_ values were defined as the drug concentration that resulted in 50% inhibition of enzyme activity.

### 4.3. Immunoblot

HUVECs (Human Umbilical Vascular Endothelial Cells) (Lonza, Walkersville, MD, USA) were cultured (24 h) in EBM-2 medium supplemented with 2% FCS. Cells were then preincubated for 30 min with various concentrations of polyphenolic compounds and subsequently stimulated with VEGF (100 ng/mL). Flavonoid concentrations were selected based on prior in vitro kinase assays. Following treatment, cells were lysed in RIPA buffer (Thermo Scientific, Waltham, MA, USA) containing Halt protease and phosphatase inhibitors, and protein concentrations were determined using the BCA assay. Minor variations in protein loading may occur when samples are prepared on different days. Equal amounts of protein were then separated by SDS–polyacrylamide gel electrophoresis, then transferred to PVDF (Polyvinylidence Fluoride) membranes. Membranes were incubated with primary antibodies against VEGFR2, phospho-VEGFR2 (Tyr1175), p44/42 MAPK, phospho-p44/42 MAPK (Thr202/Thr204) and β-actin/tubulin (loading controls) (Cell Signaling Technology, Danvers, MA, USA) followed by horseradish peroxidase (HRP)-conjugated secondary antibody and chemiluminescent substrate (Thermo Fisher Scientific, Milwaukee, WI, USA) [27,32]. Relative levels of p-VEGFR2, total VEGFR2, p-MAPK, and total MAPK were quantified by densitometric analysis of band intensities using ImageJ software version 1.54r (National Institutes of Health, Bethesda, MD, USA) and normalized to the corresponding loading control.

### 4.4. Cell Proliferation

HUVECs were used for endothelial cell proliferation assay, as described previously [32]. Endothelial cells were plated onto a gelatinized 24-well culture plate in 0.5 mL EBM-2 medium (from Lonza for HUVEC) containing 10% FCS, incubated (24 h), then treated with various concentrations of polyphenolic compounds in the absence or presence of VEGF (100 ng/mL, PeproTech Inc., Cranbury, NJ, USA, distributed by Thermo Fisher Scientific). After 48 h incubation, cells were dispersed in trypsin and counted using a Coulter counter. Results were expressed as ratio of control (vehicle treatment).

## 5. Conclusions

Flavonoids inhibit VEGFR2 activity through specific structural features, including hydroxylation patterns on the A- and B-rings and key C-ring elements. These structure–activity insights provide a basis for designing more effective and safer VEGFR2 inhibitors. Naturally derived polyphenols therefore represent promising alternatives to conventional anti-VEGF therapies with reduced adverse effects.

## Data Availability

The original contributions presented in this study are included in the article. Further inquiries can be directed to the corresponding author.

## Abbreviations

The following abbreviations are used in this manuscript:

VEGF	Vascular endothelial growth factor
SAR	Structure–activity relationship
ATP	Adenosine triphosphate
CG	Catechin gallate
ECG	Epicatechin gallate
EGCG	Epigallocatechin gallate
IC_50_	Half-maximal inhibitory concentration
MAPK/ERK2	Mitogen-activated protein kinase/extracellular signal-regulated kinase
ELISA	Enzyme-linked immunosorbent assay
